# Upper airway disease diagnosis as a predictive biomarker of therapeutic response to biologics in severe asthma

**DOI:** 10.3389/fmed.2023.1129300

**Published:** 2023-03-22

**Authors:** Sophie Cottin, Virginie Doyen, Charles Pilette

**Affiliations:** ^1^Department of Pulmonary Medicine, Cliniques Universitaires Saint-Luc, Brussels, Belgium; ^2^Department of Pulmonary Medicine, Centre Hospitalier Universitaire UCL Namur, Université catholique de Louvain, Yvoir, Belgium; ^3^Pole of Pulmonology, ENT and Dermatology, Institute of Experimental and Cliniqal Research, Université catholique de Louvain, Brussels, Belgium

**Keywords:** upper airway disease, severe asthma, nasal polyposis, predictive biomarker, theragnostics

## Abstract

Asthma is a heterogeneous disease sharing airway instability but with different biology, risk factors, and response-to-therapy patterns. Biologics have revolutionized the one-size-fits-to-all approach to personalized medicine in severe asthma (SA), which relies on the identification of biomarkers that define distinct endotypes. Thus, blood eosinophils and, to some extent, exhaled nitric oxide (FeNO) can predict the response to approved anti-type 2 (T2) biologics (anti-IgE, anti–IL-5, and anti–IL-4R alpha), whereas age at onset and comorbidities such as anxiety/depression, obesity, reflux, and upper airway disease (UAD) also influence therapeutic responses in SA. In this article, focusing on the predictive value of biomarkers for the therapeutic response to biologics in SA, we first summarize the level of prediction achieved by T2 biomarkers (blood eosinophils, FeNO) and then review whether data support the predictive value of upper airway diagnosis on such outcomes. *Post hoc* analysis of most studies with T2 biologics suggests that chronic rhinosinusitis with nasal polyps (CRSwNP) and, to a lower extent, allergic rhinitis may help in predicting clinical response. Considering that T2 biologics are now also approved for the treatment of severe CRSwNP, diagnosis of upper airway disease is a key step in determining eligibility for such therapy.

## 1. Introduction: Theragnostic biomarkers in severe asthma

Asthma is a heterogeneous disease sharing common features (e.g., airway hyperresponsiveness) but with different underlying biological (hence referred to as endotypes), risk factors, and response to treatment patterns. Most patients with asthma may be well controlled by the ICS-LABA therapy, but 5–10% of patients have a more difficult, sometimes severe disease (severe asthma, SA). The therapeutic paradigm has recently (beginning of the 21^st^ century) evolved from a one-size-fits-all approach to a phenotype-based approach that relies on the expression of specific biomarkers that are now targetable by biological therapies (1–5).

The National Institutes of Health (NIH) Biomarkers Definitions Working Group defines a biomarker as “a characteristic that is objectively measured and evaluated as an indicator of normal biological processes, pathogenic processes, or pharmacologic responses to a therapeutic intervention” (6). The WHO suggested a broader definition as “almost any measurement reflecting an interaction between a biological system and a potential hazard, which may be chemical, physical, or biological. The measured response may be functional and physiological, biochemical at the cellular level, or a molecular interaction”.^1^ There are different types of biomarkers based on their main clinical application, namely diagnosis, monitoring, pharmacodynamic/response, prediction and prognostic, safety, and susceptibility/risk assessment.^2^

The biomarkers that are currently validated in asthma relate to type 2 (T2) immunity, namely blood (or sputum) eosinophils and exhaled nitric oxide (FeNO), which are elevated in more than half of patients with severe asthma (7–9). In addition, those T2 biomarkers may overlap with allergy/atopic sensitization, which is also present in approximately half of the patients with SA, resulting in a large overlap between allergic and non-allergic patients with T2/eosinophilic asthma. Global Initiative for Asthma (GINA) guidelines recommend biologics as an add-on therapy for patients with severe T2 asthma who remain uncontrolled (including with severe exacerbations) despite step 4 therapy. Following the large overlap between T2 asthma subsets and the absence of head-to-head trials currently published with biologics that could demonstrate better efficacy in certain groups of patients (10), biomarkers that could help discriminate patients with T2 severe asthma who should be preferentially treated by an anti-T2 biologic rather than another is valuable and could partially substitute for the current practice, which is primarily based on a try-and-error approach (11–14).

Theragnostic is an emerging field of medicine that combines therapeutic and diagnostic purposes with the intention to simultaneously or sequentially diagnose and treat medical conditions. In SA, blood eosinophils and exhaled nitric oxide (FeNO) can predict the response to approved anti-T2 biologics (anti-IgE, anti–IL-5, and anti–IL-4Rα). In addition to biology, age at the onset and comorbidities, such as anxiety/depression, obesity, reflux, and upper airway disease (UAD), also influence therapeutic responses to this disease.

In this article, after summarizing the predictive value of validated T2 biomarkers, we review clinical data on the potential predictive value of upper airway disease (UAD) diagnosis on the response to biologics in SA, as well as discuss its potential positioning in the current landscape of asthma-related biomarkers.

## 2. Biomarkers predicting the response to anti-T2 therapies

Several biomarkers are available to phenotype asthma, some of which may predict clinical response to corticosteroids and T2 biologics; these include blood (or sputum) eosinophils, FeNO, serum total IgE (tIgE) levels, and periostin (15, 16).

### 2.1. Eosinophils

The prevalence of increased eosinophils is observed in ~ 50–60% of patients with mild-to-moderate asthma, and probably a larger proportion in SA (17, 18), as well as up to 80% in patients with corticosteroid-naive asthma (19).

First, airway eosinophilia is a prognostic biomarker as it correlates with the degree of airway hyperreactivity, exacerbation rate, poor symptom control, as well as small airway dysfunction (20–24). In addition, sputum eosinophilia predicts corticosteroid response in asthma, as first described by Brown in 1958 (25). This finding was confirmed 40 years later by Pavord et al. (26) and others since then (27), also showing that a treatment strategy guided by the sputum eosinophil count did reduce SA exacerbations compared to standard management (28, 29). Sputum eosinophils were considered to be more stable than blood eosinophils (30) and provided the best ROC curve among T2 biomarkers for predicting the response to a short steroid burst (31), which was confirmed by others (32). However, in the “Dose Ranging Efficacy And safety with Mepolizumab” (DREAM) study, the response to mepolizumab was poorly predicted by sputum eosinophilia ≥3% (33), in contrast to blood eosinophils, where patients with eosinophils >300/ul responded better than those with <300/ul (34, 35). It should also be noted that in the Phase I study with benralizumab, an anti–IL-5 receptor mAb, the effect of the therapy on sputum eosinophils was more variable than on blood eosinophils (36). Thus, in patients with naïve asthma, blood eosinophils correlate with sputum eosinophils with a precision ranging from 59 to 92% sensitivity and 65 to 91% specificity (37). The use of induced sputum has some limitations. First, not all patients can produce good quality sputum, and it is usually accepted that in trained teams, the success rate of the procedure (combining a successful induction and quality criteria) is approximately 70–80%. Second, sputum induction may induce bronchoconstriction, and it is recommended to administer per or pre-procedure short-acting bronchodilators as well as in case of high-risk patients (i.e., subjects with FEV1/forced vital capacity <0.7 post-salbutamol, unstable asthmatic patients or for a patient with post-bronchodilator FEV1 ≤65% predicted) to use isotonic solution instead of hypertonic saline. However, when performed according to recommendations, induced sputum is safe in subjects with moderate-to-severe asthma (32, 38, 39). Third, the procedure of sputum induction itself may influence the composition of airway inflammatory mediators for a few hours; thus, for this reason, it is advised to leave 24 h between sputum inductions to obtain reproducible results (40). Fourth, sputum eosinophils may vary over time (36, 41), and with the disease control (42), but acceptable reproducibility (Ri values > 0.8) has been reported in patients with eosinophilic inflammation (43). The predictive value of blood eosinophils was confirmed in clinical trials with reslizumab, another anti–IL-5 monoclonal antibody, with significant clinical effects observed in patients with blood eosinophils higher than 200/μl at baseline (44, 45). Accordingly, patients treated with benralizumab, an anti–IL-5 receptor mAb, and with baseline blood eosinophils ≥300/μl had significantly lower exacerbation rates (46) and a cutoff of ≥300 eosinophils/μl was used in subsequent phase III studies (47, 48). The greatest clinical benefit was observed in patients with blood eosinophil ≥150/μL and baseline FeNO of ≥25 ppb in trials with dupilumab, a mAb directed against the alpha subunit of the interleukin (IL)-4 receptor, thereby blocking both IL-4 and IL-13 (14, 49). In contrast, blood eosinophils were less predictive of clinical response to tezepelumab, an anti-epithelial TSLP antibody (50).

One important issue in clinical practice relates to the variability of blood eosinophils. Blood eosinophils may also vary due to disease activity as well as intrinsic day-to-day (and even within-day) changes. In contrast to oral steroids, which influence blood eosinophil counts, inhaled corticosteroids (ICS) impact airway eosinophils but only slightly influence blood eosinophils (51, 52). It renders mandatory repeated measurements before “labeling” a patient as eosinophilic or not (46, 53).

### 2.2. Exhaled nitric oxide (FeNO)

Nitric oxide (NO) is a gas that can be measured in exhaled breath and is increased upon the activation of the airway epithelium by IL-4/IL-13, which upregulates iNOS expression, whereas eosinophilia is primarily increased following IL-5 upregulation (54, 55). Recommendation on the exhaled biomarkers has been published by Horvath et al. (56). According to GINA and recent ERS guidelines, FeNO > 50 ppb (adults) and >35 ppb (children) are indicative of eosinophilic inflammation and the diagnosis of asthma (57). FeNO (≤20 or ≥50 ppb) may also discriminate the inflammatory phenotype (T2-low or T2-high, respectively) during asthma exacerbation (58). Similar to eosinophils, it is also a validated biomarker that predicts the response to inhaled corticosteroids (ICS) (57, 59). In addition, as iNOS is suppressed by ICS irrespectively of their effect on airway inflammation, it may help to identify non-adherence to maintenance therapy (60), as well as a persistent T2 phenotype in SA (61), and to guide the use of biological therapies (62).

Accordingly, with its regulation by IL-4/IL-13, anti–IL-5(R) biologics (mepolizumab, benralizumab) do not alter significantly FeNO. High FeNO may, however, predict a better response to mepolizumab, as “super-responders” (defined by upper 25% of ACQ-5 improvement, corresponding to 24% of patients) have a higher FeNO (41 vs. 23 ppb) at baseline (63). A stronger predictive effect was consistently observed with dupilumab, which achieved a greater benefit in patients with FeNO ≥25 ppb. In patients with a FeNO ≥50 ppb, the improvement in FEV1 reached 390 ml in the dupilumab-treated group compared to placebo (14, 64), whereas FeNO ≥25 ppb was also associated with a higher reduction of exacerbations and maintenance oral corticosteroids (OCSs) dose (64–66), as confirmed in real-life studies (67). A *post hoc* analysis found the greatest treatment response to dupilumab in patients with FeNO ≥25 ppb and blood eosinophils ≥150/μl (64). For omalizumab, the ATS/ERS guidelines recommend (conditionally and with a low quality of evidence) using a FeNO cutoff of ≥19.5 ppb to identify adolescents and adults with allergic SA who could be more likely to benefit from anti-IgE treatment (68), based on a subgroup analysis that showed a reduction of the exacerbation rate, a longer time to first asthma exacerbation and a larger improvement of mean QLQ in the ≥19.5 ppb subgroup (69). In contrast, the benefit of tezepelumab on exacerbations was observed irrespectively of baseline FeNO (50).

### 2.3. Total serum IgE

The first biologic that was developed and demonstrated efficacy in severe asthma is omalizumab (1–4), which is a humanized anti-IgE mAb that binds the C3 region of the IgE-Fc fragment and captures circulating IgE, preventing interaction with the FcεRI and thus interrupting the allergic cascade (70). Omalizumab reduced asthma exacerbations, inhaled corticosteroid dose, rescued medication use, as well as the rate of serious asthma exacerbations and the need for unscheduled outpatient visits, emergency room treatment, and hospitalization in patients with moderate-to-severe allergic asthma (71). Baseline tIgE is a poor predictor of anti-IgE efficacy, the only impact being observed in the pivotal INNOVATE study (72) is that patients in the lowest quartile (i.e., <76 kU/L) had a significantly lower benefit. Pooled analysis showed that the benefits were globally irrespective of total IgE levels and that pre-treatment baseline characteristics cannot reliably predict which patients will benefit the most from omalizumab (11, 73). However, a more recent study showed that T2 biomarkers (FeNO, eosinophils, and periostin) could slightly but significantly predict a better response to omalizumab (69).

### 2.4. Periostin and other biomarkers

Periostin is an extracellular matrix protein produced by airway epithelial cells and fibroblasts upon activation by IL-4 or IL-13, which is implicated in tissue remodeling (74) and correlated with lung function decline (75) as well as asthma exacerbations (despite high-dose ICS therapy). Patients with high serum levels of periostin had a greater improvement in lung function and reduced asthma exacerbations following treatment with the anti–IL-13 lebrikizumab (76–78). Other biomarkers, such as eosinophil cationic protein (ECP), eosinophil-derived neurotoxin (EDN), galectin-10, or bromotyrosine (BrTyr), have also been studied, as well as volatile organic compounds (VOCs) but are not yet validated and/or implemented for use in clinical practice (79–91).

In addition to markers related to airway immunobiology, late (adult) vs. early (childhood) onset of the disease is associated with non-allergic, eosinophilic vs. allergic asthma, respectively (5). Subsequently, adult disease onset in eosinophilic SA may be considered a clinical biomarker that could predict an enhanced role of eosinophilic, rather than allergic, inflammation.

## 3. Upper airway disease as a theragnostic biomarker in severe asthma

Upper airway diseases (UADs) are mainly represented by allergic rhinitis (and rhinoconjunctivitis), non-allergic rhinitis, and chronic rhinosinusitis with (CRSwNP) or without nasal polyps (CRSsNP), all of which are highly prevalent comorbidities that impact disease control in asthma. Thus, it is estimated that 80% of patients with asthma have allergic rhinitis, while 22–42% have CRS (92). Similarly to asthma, different phenotypes of CRSwNP have been described. Most patients with CRSwNP show a T2 inflammation, as shown by a recent study in which 87% of patients with CRSwNP had T2 inflammation, while only a few are characterized by T1 or T3 inflammation or a mixed phenotype (17, 18, and 26%, respectively) (93). The currently approved biologics for asthma target the T2 immune pathway. Pathogens and environmental factors may induce, following interactions with the airway epithelium and antigen-presenting cells, the differentiation of naïve CD4^+^ T cells into Th2 cells that release IL-4 and IL-13 interacting with B cells to produce IgE that may activate mast cells. IL-5 is also released by Th2 and innate lymphoid cell (ILC)-type 2, acting as a pivotal factor in the differentiation, survival, and activation of eosinophils, basophils, and mast cells (94).

Data on the potential predictive value of upper airway disease (UAD) diagnosis on the biologic response in severe asthma emerged from *post hoc* analyses of randomized, placebo-controlled trials (RCTs) by stratifying *a posteriori* enrolled patients according to the presence (vs. absence) of UAD, specifically allergic rhinitis or CRSwNP. This will be discussed separately for each biologic that is currently approved for the treatment of SA. Interestingly, the same biologics—namely anti-IgE, anti–IL-5, and anti–IL-4Rα–have been recently approved for the treatment of severe CRSwNP (while dupilumab was approved first in severe CRSwNP, before asthma), further increasing the relevance of integrating UAD in the management of (severe) asthma.

### 3.1. Omalizumab

Omalizumab is the first monoclonal antibody registered (in 2003) for asthma treatment, before other indications such as chronic rhinosinusitis with nasal polyposis (CRSwNS) and chronic urticaria. Few studies evaluated the impact of allergic rhinitis as a biomarker of response to omalizumab. One *post hoc* analysis of a phase 3 RCT conducted in Chinese patients with moderate-to-severe persistent allergic asthma showed that asthma symptoms (ACQ score) and asthma-related quality of life (QLQ score) were significantly improved after 24 weeks of treatment with omalizumab compared to placebo in patients with perennial allergic rhinitis, in contrast with those without rhinitis. Unfortunately, the level of blood eosinophils according to the presence (or not) of rhinitis was not reported; the mean level is 296/μl in the total cohort (95) (Table 1). In another retrospective study of the reversal of airway obstruction (defined by FEV1 normalization, vs. persistent airflow limitation, PAL) upon omalizumab in severe allergic asthma (up to 4 years of treatment), patients with FEV1 normalization had a significantly higher proportion of rhinitis than patients with PAL (83 vs. 43%; *p* = 0.027). The same finding was seen when considering CRSwNP, 72% of patients with FEV1 normalization had CRSwNP compared to 29% in the PAL group (*p* = 0.031). It is important to notice that the mean levels of blood eosinophils and FeNO were higher in the group of patients with reversal of airway obstruction than in those with persistent obstruction (754/μL and 66.8 ppb vs. 351/μL and 23 ppb, respectively) (97).

**Table 1:** Predictive value of UAD diagnosis on asthma-related outcomes in *post hoc* analysis of randomized controlled trials [except ref. (88–90, 96)] with biologics.

**Treatment**	**Duration of treatment (n patients)**	**Outcome parameter**	**Effect of treatment, in patients with or without UAD**	**References**
Omalizumab	24 weeks (*n =* 608)	ACQ-5	Improvement (least squares mean difference) vs. placebo, significant in patients with (−0.4; *p =* 0.009) but not without (-0.2; *p =* 0.07) persistent AR.	(95)
	QLQ		Improvement (least squares mean difference) vs. placebo significant in patients with (+0.7; *p =* 0.001) but not without (+0.3; *p =* 0.054) persistent AR.	
	Up to 48 months (*n =* 32)	Normalization of lung function (FEV1)	Among patients “normalized”, 15 patients (83%) had rhinitis while 6 patients (43%) with persistent airflow limitation had rhinitis (*p =* 0.027; OR 6.7).	(97)
			Among patients “normalized”, 13 patients (72%) had CRSwNP, while 4 patients (29%) with persistent airflow limitation had CRSwNP (*p =* 0.031; OR 6.5).	
	6 months (*n =* 180)	ACQ-5	Mean difference vs. baseline, −1.8 (*p < * 0.0001) in patients with CRSwNP and −1.6 (*p < * 0.0001) in patients without CRSwNP.	(98)
		FEV1, % predicted (post-bronchodilation)	Mean difference vs. baseline, +12.6% (*p =* 0.578) in patients with CRSwNP and +3.8% (*p =* 0.194) in patients without cRSwNP.	
Mepolizumab	52 weeks (*n =* 99)	Responder status (defined by a 350% reduction in SAE or 350% reduction of mOCS dose)	CRSwNP in 39 (54%) responders, vs. in 7 (26%) non-responders (*p =* 0.012)	(99)
		Super-responder status (defined as absence of exacerbation throughout the study and discontinuation of mOCS)	CRSwNP in 19 (68%) super-responders, vs. 7 (26%) non-responders (*p =* 0.007).	
	32 weeks (MENSA) 24 weeks (MUSCA) *Post hoc* of MUSCA (*n =* 551) Meta-analysis of MUSCA and MENSA (*n =* 936)	HRQOL (St George's respiratory questionnaire) SAE annuel rate (asthma worsening requiring systemic corticosteroids and/or hos- pitalization, and/or emergency room visit	MUSCA, *post hoc*: improvement of 14.6% (SGRQ) in patients with CRSwNP, vs. 6.5% of patients without CRSwNP. MUSCA and MENSA meta-analysis: improvement of 80% (SAE reduction) in patients with CRSwNP vs. 49% in patients without cRSwNP.	(100)
	24 weeks (SIRIUS and MUSCA), 32 weeks (MENSA) and 52 weeks (DREAM) (*n =* 1,878)	Exacerbations (defined as worsening of asthma that required the use of systemic corticosteroids and/or hospitalization/emergency room visits)	Greater effect on the rate of exacerbations in patients with vs. patients without CRSwNP: RR 0.32 vs. 0.56; *p =* 0.001. No difference on the rate of exacerbations in patients with vs. patients without AR: RR 0.50 vs. 0.50; *p =* 0.854	(96)
		ACQ-5	Greater change from baseline of ACQ-5 in patients with vs. patients without CRSwNP: −0,57 vs. −0.28; *p =* 0.051. No difference on the change from baseline of ACQ-5 in patients with AR vs. patients without AR: respectively, −0.32 vs. −0.32; *p =* 0.530	
		St George's Respiratory Questionnaire	Trend to greater change from baseline of SGRQ in patients with vs. patients without CRSwNP: −11.3 vs. −6.0; *p =* 0.063. No difference on the change from baseline of SGRQ in patients with vs. patients without AR: respectively −8.0 vs. −6.1; *p =* 0.358	
		Pre-bronchodilator FEV1 (ml)	Greater change of FEV1 from baseline in patients with vs. patients without CRSwNP: respectively, +286.9 vs. +27.1 mL; *p < * 0.001. Trend to lower change of FEV1 from baseline in patients with vs. patients without AR: +45.9 vs. +99.8 mL; *p =* 0.099	
Reslizumab	52 weeks (*n =* 953)	Exacerbations (defined by use of systemic corticosteroids or doubling SCS dose for ≥3 days in mOCS patients, or emergency department visit or hospitalization or unscheduled physician visit)	In the OCS-dependent group, greater reduction of AE in patients with CRSwNP vs. patients without CRSwNP (rate ratio treatment/placebo not reported; *p =* 0.343).	(101)
	15 weeks (*n =* 106)	ACQ-5	Mean change from baseline, −1.0 with reslizumab vs −0.1 with placebo (*p =* 0.0119) in patients with CRSwNP; −0.5 with reslizumab vs. −0.4 with placebo (*p =* 0.7) in patients without CRSwNP.	(44)
		FEV1, absolute value (L)	Mean change from baseline, + 0.18 L with reslizumab vs. −0.04 L with placebo (*p =* 0.0625) in patients with CRSwNP; + 0.18 L with reslizumab vs. +0.1 L with placebo (*p =* 0.0257) in patients without CRSwNP.	
	52 weeks (*n =* 953)	“Super-responder” (defined by ≥10% FEV_1_ or ≥5% predicted FEV_1_ improvement, no AE throughout study, or >1.5 improvement ACQ-6	Out of 80 super-responders, a higher proportion had CRSwNP (abstract content).	(102)
	52 weeks (*n =* 953)	Annual rate of AE, defined by (at least one criteria met): Use of SCS or increase in ICS, or increase > doubling mOCS for >3 days; or unscheduled visit or ER for asthma, or hospitalization.	Reduction of 83% vs. placebo in patients with CRSwNP (RR 0.17, 95% CI 0.10–0.32; *p =* 0.0002); 70% vs. placebo in patients without CRSwNP (RR 0.30, 95% CI 0.20–0.44; *p =* 0.0103).	(103)
Benralizumab	48 weeks (SIROCCO) and 56 weeks (CALIMA) (*n =* 2,295)	FEV1	Improvement only significant in patients with CRSwNP, regardless of the administration scheme (4 or 8 wks interval) (*graphical content*).	(13)
		AE annual rate (exacerbation defined by initiation or temporary increase of SCS for ≥3 days, or single CS depot injection, emergency visit or hospitalization)	Improvement only significant in patients with CRSwNP, regardless of the administration scheme (*graphical content)*	
	48 weeks (SIROCCO) and 56 weeks (CALIMA) (*n =* 2,295)	AE annual rate (exacerbation defined by initiation or temporary increase of SCS for ≥3 days, or a singleinjection of depotCS, emergenc visit or hospitalization)	Overall population (benralizumab every 8 weeks): reduction, rate ratio vs. placebo, 0.5 (*p < * 0.001) in patients with CRSwNP; 0.68 (*p < * 0.001) in patients without CRSwNP. In the ≥300/μL blood eosinophils group: reduction, rate ratio vs. placebo, 0.46 (*p < * 0.001) in patients with CRSwNP; 0.62 (*p < * 0.001) in patients without CRSwNP. In the < 300/μL blood eosinophils group: reduction, rate ratio vs. placebo, 0.49 (*p =* 0.115) in patients with CRSwNP; 0.71 (*p =* 0.015) in patients without CRSwNP.	(104)
		FEV1 change (L), prebronchodilation	Overall population (benralizumab every 8 weeks): Improvement, LS mean difference vs. placebo, 0.29L (*p < * 0.001) in patients with CRSwNP; 0.06 (*p =* 0.032) in patients without CRSwNP. In the ≥300 /μL blood eosinophils group: Improvement, LS mean difference vs. placebo, 0.27L (*p < * 0.001) in patients with CRSwNP; 0.10 (*p =* 0.003) in patients without CRSwNP. In the < 300 /μL blood eosinophils group: LS mean difference vs. placebo, 0.24L (*p =* 0.045) in patients with CRSwNP; 0.02 (*p =* 0.581) in patients without CRSwNP.	
		Total Asthma Symptom Score (composite, daytime and night-time symptoms scored 0–6 points)	Overall population (benralizumab every 8 weeks): improvement, LS mean difference vs. placebo, −0.35 (*p =* 0.005) in patients with CRSwNP; −0.14 (*p =* 0.019) in patients without CRSwNP. In the ≥300/μL blood eosinophils group:): improvement, LS mean difference vs. placebo, −0.30 (*p =* 0.029) in patients with CRSwNP; −0.22 (*p =* 0.003) in patients without CRSwNP. In the < 300/μL blood eosinophils group): improvement, LS mean difference vs. placebo, −0.46 (*p =* 0.107) in patients with CRSwNP; −0.11 (*p =* 0.265) in patients without CRSwNP.	
		ACQ-6	Overall population: improvement, LS mean difference vs. placebo, −0.38 (*p =* 0.002) in patients with CRSwNP; −0.20 (*p < * 0.001) in patients without CRSwNP. In the ≥300/μL blood eosinophils group: improvement, LS mean difference vs. placebo, −0.39 (*p =* 0.005) in patients with CRSwNP; −0.27 (*p < * 0.001) in patients without CRSwNP. In the < 300/μL blood eosinophils group: improvement, LS mean difference vs. placebo, −0.24 (*p =* 0.416) in patients with CRSwNP; −0.17 (*p =* 0.069) in patients without CRSwNP.	
Mepolizumab, reslizumab, benralizumab	2 years (*n =* 114)	Super-responders (defined by no mOCS, no OCS bursts in the past 3 months, ACQ < 1.5, FEV1 80%, FeNO < 50 ppb, complete control of comorbidities). Non-responders (defined by discontinuation of anti-IL-5 after < 2 years because of increased symptoms, decreased FEV1 or increased OCS use). Partial responders (patients who did not fulfill the above criteria)	6% of super-responders had CRSwNP at baseline, vs. 25% of non-responders and 26% of partial responders (*p =* 0.112); 19% of super-responders had allergic rhinoconjunctivitis at baseline, vs. 33% of non-responders and 26% of partial responders (p = 0.150).	(105)
Dupilumab	12 months (*n =* 64)	FEV1 (ml)	After 12 months of treatment, median FEV1 (ml) increase was 2,400 in patients with CRSwNP vs. 1.810 in patients without CRswNP(*p =* 0.032).	(105)

AR, allergic rhinitis; mOCS, maintenance oral corticosteroids; (S)AE, (severe) asthma exacerbation.

In an Australian prospective observational study of patients with allergic SA, 15% had CRSwNP, and the benefit of omalizumab on asthma symptoms (ACQ-5 score) and lung function (FEV1% predicted) after 6 months of treatment was similar whether or not patients had NP (ACQ-5 mean difference −1.6 without vs. −1.8 with CRSwNP; FEV1 change +3.8% without vs. +12.6% with CRSwNP) (98) (Table 1).

### 3.2. Mepolizumab

Mepolizumab was the first registered (in 2004) anti–IL-5 biologic for the treatment of severe eosinophilic asthma (SEA), before validation in other conditions including CRSwNP, hypereosinophilic syndrome (HES), and eosinophilic granulomatosis with polyangiitis (EGPA). A few studies showed that CRSwNP may predict the response to mepolizumab in SA. A first retrospective single-center study of patients who received mepolizumab for SEA (>300/μL in the preceding 12 months) and classified as responders [defined by a ≥50% reduction in exacerbations, or ≥50% reduction of the prednisolone dose if requiring maintenance oral corticosteroids (mOCS)], super-responders (absence of exacerbation for the 52 weeks of follow-up and discontinuation of mOCS), or non-responders showed that CRSwNP at baseline was associated with the responder and super-responder status. Indeed, 54.2% of responders and 67.9% of super-responders had CRSwNP compared to 25.9% of non-responders (*p* = 0.012 and *p* = 0.007, respectively) (99) (Table 1).

In a *post hoc* analysis of the MUSCA trial, mepolizumab had a greater benefit on health-related quality of life-related to lower airway symptoms in patients with SEA and CRSwNP compared to patients without NP, whereas, in a meta-analysis of MUSCA and MENSA trials, the benefit of mepolizumab on the annual exacerbation rate was also greater in patients with CRSwNP compared to patients without CRSwNP (80% reduction in patients with, vs. 49% in patients without CRSwNP). In this last meta-analysis, patients with CRSwNP had a higher mean blood eosinophil count than those without CRSwNP (440/μl vs. 209/μl) (100). A *post hoc* meta-analysis of the DREAM, MENSA, SIRIUS, and MUSCA studies also reports a superior effect of mepolizumab in SEA patients with UAD. Indeed, the presence of nasal polyps (compared to the absence of CRSwNP) was associated with a significantly higher benefit in terms of exacerbations (RR 0.32 vs. 0.56; *p* = 0,001) and change in FEV1 from baseline (+286.9 ml vs. + 27.1; *p* = 0,001). A trend favoring mepolizumab (vs. placebo) in patients with CRSwNP was shown when evaluating effects on ACQ-5 and St George's Respiratory Questionnaire. The presence of allergic rhinitis was also evaluated as a predictor of response but showed no beneficial effect in terms of exacerbations, FEV1, ACQ-5, or on the St George's Respiratory Questionnaire (Table 1). Mean levels of blood eosinophils across the four studies were from 250 to 340/μl (96).

### 3.3. Reslizumab

Reslizumab, a humanized IgG4 monoclonal antibody that targets IL-5, was the second anti–IL-5 biologic registered for SEA (in 2017). A *post hoc* analysis of two duplicate RCTs in which patients with eosinophilic (≥400 cells/μL) asthma and who remained inadequately controlled on at least medium-dose ICS were randomized to placebo or reslizumab (3 mg/kg every 4 weeks) for 52 weeks and categorized by use of maintenance OCS. As a secondary outcome, the *post hoc* analysis assessed biomarkers that predicted response in terms of exacerbations (defined by the use of systemic corticosteroids in steroid-naïve patients or a doubling of systemic corticosteroids dose for ≥3 days in OCS-dependent patients, emergency department visit, or hospitalization, or unscheduled physician visit) in OCS-dependent patients and showed that the presence of CRSwNP did not impact the benefit on asthma exacerbations, although there was a trend toward higher reduction in patients with CRSwNP. The mean blood eosinophil count was 607/μl in those patients at baseline, but blood eosinophil counts in patients with or without CRSwNP were not reported (101) (Table 1).

The benefit of reslizumab on asthma symptoms (ACQ-5 score) was significant in patients with CRSwNP (−1.0 vs. −0.1 in the placebo group, *p* = 0.012), whereas only a trend was observed for the overall population (−0.7 vs. −0.3, *p* = 0.054). In contrast, the benefit of lung function was not increased in patients with CRSwNP. The median blood eosinophil count was 600/μl in the CRSwNP group and 400/μl in the group without CRSwNP (44). Another *post hoc* analysis of the same phase 3 trials, classified patients as non-responders, moderate-responders, high-responders, or super-responders based on the number of the following criteria at week 52 (0, 1, 2, or 3, respectively): ≥10% FEV1 improvement, or ≥5% predicted FEV1 improvement, no exacerbation during the 52 weeks of study, or >1.5 improvements of ACQ-6. They found that compared to non-responders, super-responders to reslizumab were more likely to have CRSwNP (102).

A recent *post hoc* study showed that the reduction in the annual exacerbation rate was almost similar between groups, with a slightly higher percentage in patients with CRSwNP (83 vs. 70%) (103).

### 3.4. Benralizumab

Benralizumab, the only approved humanized anti–IL-5 receptor antibody, was also approved for SEA treatment in 2017 following SIROCCO and CALIMA phase 3 trials in which patients with severe, uncontrolled asthma receiving high-dose ICS plus LABA received either benralizumab every 4 or 8 weeks, or placebo. Evaluation of treatment was made up to 48 weeks in the SIROCCO study and up to 56 weeks in the CALIMA study (47, 48). A first study showed that a greater improvement in FEV1 and asthma exacerbation rate was reached in patients with CRSwNP, in addition to those with blood eosinophils ≥300 cells/μl, and regardless of the administration scheme of benralizumab (every 4 or 8 weeks) (13) (Table 1). In the second *post hoc* analysis of the SIROCCO and CALIMA trials on annual exacerbation rate in the overall population and in the ≥300/μl blood eosinophil population, the presence of CRSwNP increased the benefit of benralizumab every 8 weeks vs. placebo compared to the overall population, with CRSwNP ranking second as a predictive factor after OCS use at baseline. In patients with <300 eosinophils/μl, the effect of CRSwNP was not significant although a trend to a higher reduction of AER was observed. Similar findings were observed for lung function (pre-bronchodilation FEV1), with the greatest impact of CRSwNP observed with benralizumab vs. placebo. Similar results were also achieved in the ≥300 eosinophils/μl population and for asthma symptoms, compared to the overall population (104) (Table 1).

Finally, one single-center, real-life study reported that patients with CRSwNP or allergic rhinoconjunctivitis had a lower chance of being super-responders to anti–IL-5(R) biologics (mepolizumab, reslizumab, or benralizumab), but this was probably biased by the fact that complete control of comorbidities (chronic rhinosinusitis and NP, as well as chronic otitis, allergic rhinoconjunctivitis, and atopic dermatitis) was required to define such response (Table 1). In this population, FeNO levels at baseline were similar across the groups of patients (non-responders, partial responders, and super-responders), and all groups showed a blood eosinophil count >300/μl, with the highest level in partial responders (570/μl) (105).

### 3.5. Dupilumab

Dupilumab is a monoclonal antibody targeting IL-4Rα and subsequently inhibiting IL-4 and IL-13 activities. It was recently approved (in 2019) for asthma treatment, and it is also indicated in severe CRSwNP and severe atopic dermatitis. However, only a few data are available to assess whether UAD may predict response to dupilumab in asthma. A French study reported a greater improvement in lung function in patients with CRSwNP than those without (Table 1). In contrast, blood eosinophils did not significantly predict the effect on lung function, OCS use, or ACT score (using 100/μ or 300/μ cutoffs) (105).

## 4. Conclusion

Blood eosinophils and FeNO have emerged as biomarkers capable of predicting the response of patients with asthma to ICS and T2 biologics. They are easy to measure, in both in- and out-patient settings and are non-invasive, but they suffer from variability (mainly for eosinophils, e.g., between-sample or nyctemeral) and/or multiple interfering factors such as environmental exposures, e.g., to cigarette smoke or allergens (mainly for FeNO). This article provides a comprehensive review of the data collectively indicating that UAD, in particular, CRSwNP and, to a lower extent, allergic rhinitis, predict the response to T2 biologics in severe asthma, as well as being improved by such therapies. Moreover, CRSwNP could be viewed as a stable biomarker that does not suffer from intrinsic variability, at least in Western countries, as CRSwNP displays a different inflammatory phenotype in Eastern countries (106). Since most studies did not specifically address this point, it is unclear to what extent the prediction by CRSwNP status is independent (or not) of eosinophils or FeNO. In contrast, the efficacy of anti–IL-5 biologics on CRSwNP outcomes (such as the polyp size) does not seem to differ whether patients had comorbid asthma or not (107). This observation is consistent with the fact that CRSwNP is most often eosinophilic/T2 in nature (in Western populations), whereas the underlying biology of SA may be related more frequently to different endotypes.

**Figure 1:**
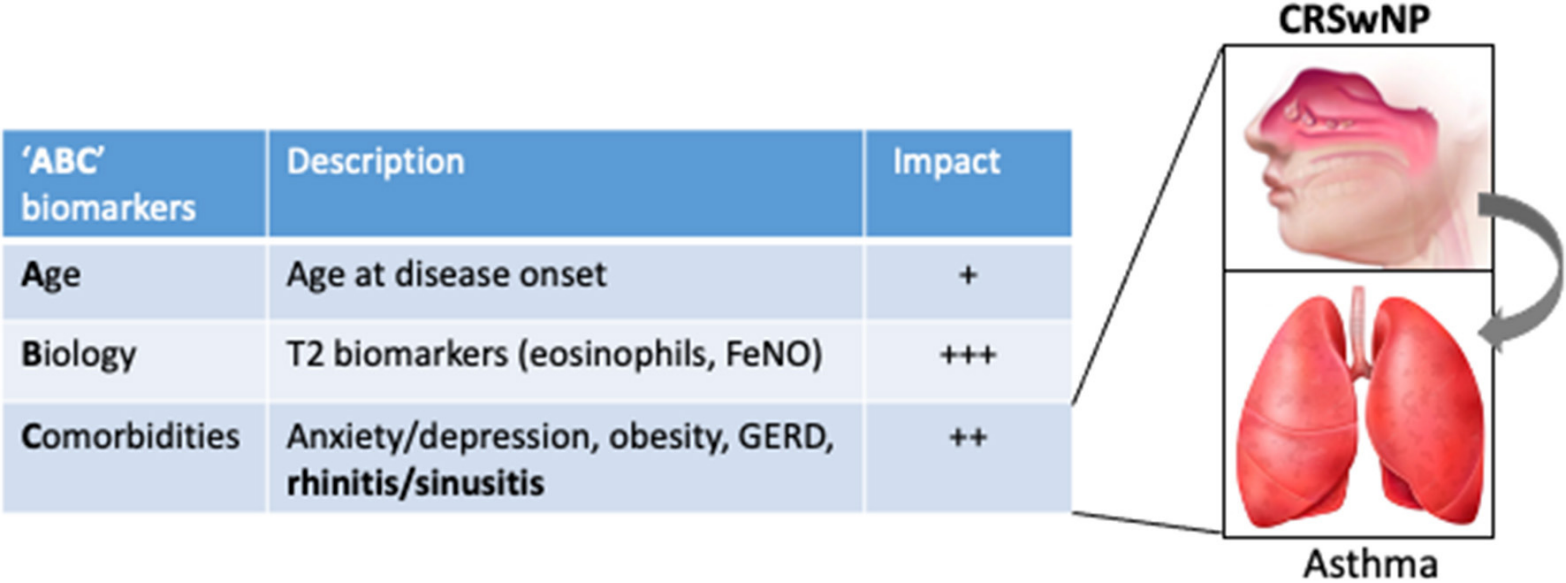
The “ABC” sets of biomarkers that influence the response to biologics in severe asthma, illustrated by their respective estimated impact on the response to anti–IL-5 therapies. For instance, the impact calculated (odds ratio, OR) for adult onset and CRSwNP on the probability to be defined as a responder to mepolizumab in a “real-life” series (88) were 1.20 and 1.36 (vs. the absence of CRSwNP), while it was not significant for eosinophils. *Inset*, illustrating the impact of UAD (in particular CRSwNP) on asthma outcomes upon biotherapy.

Altogether the data reviewed in this article support that the diagnosis of UAD in patients with SA with the indication of biotherapy is a key step in refining the clinical profile and has added value to T2 biomarkers (blood eosinophils and FeNO) to predict the clinical response. As there is no published head-to-head trial comparing biologics for CRSwNP or for severe asthma and, although some studies are ongoing (10), only indirect comparisons are available, we propose to refer to the acronym “ABC,” standing for age (at the onset of the disease), biology (eosinophils, FeNO), and comorbidities, as the three key sets of biomarkers that impact the therapeutic response to biologics in SA (Figure 1) and that could help clinicians to decide the first most appropriate biologic in a given patient. In particular, UAD represents a strong factor with comorbid CRSwNP-SA, defining a particular eosinophilic condition for which anti–IL-5 biologics have increased efficacy and should be managed through a collaborative and multidisciplinary approach (108).

## Author contributions

SC and VD drafted the manuscript. CP designed and revised the manuscript and made the figure. All authors contributed to the article and approved the submitted version.

## Footnotes

^1^Inchem. Available online at: http://www.inchem.org/documents/ehc/ehc/ehc155.htm.

^2^Manzanares. Available online at: https://www.sciencedirect.com/topics/agricultural-and-biological-sciences/biomarkers.
